# Response to the letter to the editor regarding: “A call for dynamic guidelines: Incorporating AI ethics into social media best practices for spine professionals”

**DOI:** 10.1016/j.xnsj.2026.100856

**Published:** 2026-01-27

**Authors:** Zachary A. Cupler, Andrew Trontis, Brandon Lucke-Wold, Samuel M. Schut, Khoi D. Than, James E. Eubanks, Robert J. Butler, Reem Elwy, David Gendelberg

**Affiliations:** a Physical Medicine and Rehabilitative Services, Butler VA Health Care System, Butler, PA, United States; b Progressive Spine and Orthopedics, Englewood, NJ, United States; c Department of Neurosurgery, University of Florida, Gainesville, FL, United States; d Department of Wellness and Preventive Medicine, Cleveland Clinic, Cleveland, OH, United States; e Center for Wellness Research and Training, Cleveland Clinic, Cleveland, OH, United States; f Cleveland Clinic Lerner College of Medicine of Case Western Reserve University School of Medicine, Cleveland, OH, United States; g Department of Neurosurgery, Duke University, Durham, NC, United States; h Department of Orthopedics and Physical Medicine and Rehabilitation, Division of Physical Medicine and Rehabilitation, Medical University of South Carolina, Charleston, SC, United States; i Department of Physical Medicine and Rehabilitation, University of Pittsburgh Medical Center, Pittsburgh, PA, United States; j Integrated Primary Care, VA Palo Alto Health Care System, Palo Alto, CA, United States; k Cairo University, Cairo, Egypt; l Department of Orthopaedic Surgery, Orthopaedic Trauma Institute, University of California San Francisco, San Francisco, CA, United States

To the Editor,

We thank Sham et al. for their persuasive letter to the editor [1] pertaining to our recent article, ``Social Media Best Practices for the Spine Care Professional,” [2] with regards to responsible and ethical use of artificial intelligence (AI). The authors propose strong rationale to incorporate AI when considering future iteration of social media best practice guidelines for spine care professionals. Their proposed AI recommendations for future guidelines focused on three themes: (1) accuracy and validity of AI-generated output as guided by a human, (2) transparency and trust to the public by ensuring appropriate disclosure of AI-reliance to generate or modify content, and (3) attribution and originality to identify source material, avoid misattributions, and prevent unsupervised thought being conveyed as clinical opinion.

Identified as out of scope of our paper in our limitations, we do recognize the importance of AI in future guidelines and highlighted that we anticipated future iterations to these best practices as new technology emerges which rightfully should consider AI in this context. We are pleased to read such positive feedback regarding our synthesis of best practices for social media use for spine care professional. Individual members of this authorship team maintain interest in AI initiatives. The North American Spine Society does have workgroups with particular interest in AI that are prioritizing guideline development for application among spine care professionals.

## Disclaimers

This is not an official position of the North American Spine Society.

The views expressed in this article are those of the authors and do not reflect the official policy or position of the Department of Veterans Affairs, or the United States Government.

## Declaration of Competing Interest

The authors declare that they have no known competing financial interests or personal relationships that could have appeared to influence the work reported in this paper. All authors acknowledge their personal use of social media platforms.
